# Pyruvate dehydrogenase kinase 1 interferes with glucose metabolism reprogramming and mitochondrial quality control to aggravate stress damage in cancer

**DOI:** 10.7150/jca.34330

**Published:** 2020-01-01

**Authors:** Xinyue Deng, Quan Wang, Meiyu Cheng, Yingying Chen, Xiaoyu Yan, Rui Guo, Liankun Sun, Yang Li, Yanan Liu

**Affiliations:** 1 Department of Pathophysiology, College of Basic Medical Sciences, Jilin University, Changchun, Jilin, China.; 2 Department of Radiation Oncology, China-Japan Union Hospital of Jilin University, Changchun, Jilin, China.

**Keywords:** DCA, glucose metabolic reprogramming, mitochondiral quality control, oxidative phosphorylation, PDK1.

## Abstract

Pyruvate dehydrogenase kinase 1 (PDK1) is a key factor in the connection between glycolysis and the tricarboxylic acid cycle. Restoring the mitochondrial OXPHOS function by inhibiting glycolysis through targeting PDK1 has become a hot spot for tumor therapy. However, the specific mechanism by which metabolic changes affect mitochondrial function remains unclear. Recent studies have found that mitochondrial quality control such as mitochondrial protein homeostasis plays an important role in maintaining mitochondrial function. Here, we focused on PDK1 and explored the specific mechanism by which metabolic changes affect mitochondrial OXPHOS function. We showed that glucose metabolism in HepG2 and HepG3B cells switched from anaerobic glycolysis to the mitochondrial tricarboxylic acid cycle under different concentrations of dichloroacetate (DCA) or short hairpin PDK1. After DCA treatment or knockdown of PDK1, the mitochondrial morphology was gradually condensed and exhibited shorter and more fragmented filaments. Additionally, expression of the mitochondrial autophagy proteins parkin and PTEN-induced kinase was down-regulated, and the biosynthetic protein peroxisome proliferator-activated receptor gamma coactivator 1α (PGC1α) and its regulated complex I, III, IV, and V protein were down-regulated. This indicated that PDK1 inhibition affected the level of mitochondrial quality control. Analysis of mitochondrial function revealed significantly increased mitochondrial reactive oxygen species and decreased membrane potential. Therefore, glucose metabolism reprogramming by PDK1 inhibition could induce mitochondrial quality control disorders to aggravate mitochondrial stress damage.

## Introduction

Tumor cells have a unique glycolysis phenotype (also known as the Warburg effect), which typically show increased glycolysis and reduced mitochondrial oxidation regardless of the availability of oxygen. This metabolic reprogramming of tumor cells constitutes an enormous advantage for tumor growth and contributes to apoptosis resistance 1, 2. Previous studies found that reversing the Warburg phenotype of tumor cells promoted the recovery of mitochondrial function and apoptosis to improve therapeutic effects. Mitochondrial function plays an important role in tumor survival, and recent studies have targeted mitochondria as a new strategy for cancer chemotherapy 3, 4. However, the mechanisms of how changes in metabolic patterns affect tumor cell mitochondrial function are unclear. Mitochondrial function, especially the normal functioning of OXPHOS, requires the interaction of multiple proteins to support the integrity of cell organelles 5, 6. Therefore, the homeostasis of mitochondrial proteins is particularly important for cell survival. As such the influence of metabolic changes on mitochondrial protein homeostasis and specific regulatory mechanisms has become the focus of our research.

Pyruvate dehydrogenase kinase 1 (PDK1), a gatekeeper of glycolysis and mitochondrial OXPHOS, has attracted increasing attention for the regulation of tumor metabolism 7. PDK1 phosphorylates pyruvate dehydrogenase (PDH) to inhibit its activity, thereby reducing the level pyruvic acid in the tricarboxylic acid cycle, which affects the rates of OXPHOS 8. PDK1 inhibition activates OXPHOS and the electron transport chain, resulting in increased phosphorylation byproducts such as reactive oxygen species (ROS), which restores apoptosis in the mitochondrial pathway 9. This suggests that exploring the role of PDK1 might explain the role of mitochondria in tumor cells.

Quality control of mitochondria is a prerequisite for maintaining mitochondrial oxidative phosphorylation. Mitochondrial quality mainly includes biosynthesis, dynamics, autophagy, and control of mitochondria protein homeostasis 6, 10, 11. Peroxisome proliferator-activated receptor gamma co-activator (PGC)-1α is the main protein of mitochondrial biosynthesis and has an important role in regulating energy metabolism 12-14. It is located downstream is mitochondrial transcription factor A (Tfam), which controls the transcription of mitochondrial DNA-encoded complexes that in turn regulate the mitochondrial OXPHOS function^15^, ^16^. Mitochondria are constantly undergoing fusion and fission, which is necessary to maintain mitochondrial OXPHOS function and homeostasis 17-19. Mitochondrial dynamics is closely related to mitochondrial autophagy, and the segregation of terminally damaged mitochondria enables degradation by selective autophagy termed mitophagy 20, 21. Additionally, the mitochondrial unfolded protein response (UPR) is a stress response that activates heat shock protein (HSP) family proteins to promote protein homeostasis within the organelle 6, 22. Interestingly, the induction of mitochondrial biogenesis could activate UPR^mt^ 23, suggesting that mitochondrial quality control plays an important role in maintaining mitochondrial function.

Dichloroacetate (DCA), a PDK1 inhibitor, reverses the metabolic phenotype of tumor cells, shifting the metabolic flux to mitochondrial oxidative phosphorylation, which induces selective cytotoxicity 24-26. Recently, Chaudhary et al. reported that inhibition of PDK1 induced mitochondrial UPR responses in LNCaP cells 27. In a neuroblastoma study, Pajuelo-Reguera et al. found that PDK1 inhibition altered the mitochondrial network morphology of tumor cells by regulating mitochondrial fusion, thereby inhibiting tumor cell proliferation and promoting apoptosis 28. This suggests that PDK1 may play a regulatory role in regulating metabolism and mitochondrial quality control.

In this study, the relationship between PDK1-induced altered glucose metabolism and mitochondrial quality control was investigated by studying PDK1 as an entry point in tumor cells. We demonstrated that glucose metabolism reprogramming by PDK1 inhibition could induce mitochondrial quality control disorders to aggravate mitochondrial stress damage, which provides new targets and ideas for the clinical treatment of tumors.

## Methods and materials

### Reagents and antibodies

The human liver cancer cell lines HepG2 and HepG3B were obtained from the cell bank of the Institute of Biochemistry and Cell Biology (Shanghai, China). Both cell types were cultured in DMEM medium (Gibco, Carlsbad, CA). The glycolysis inhibitor DCA, 3-(4, 5-dimethylthiazol-2-yl)-2,5-diphenyltetrazolium bromide (MTT) and N-acetyl-L-cysteine (NAC) were purchased from Sigma-Aldrich (St Louis, MO). Anti-dynamin-related protein (DRP1), anti-mitofusin 2 (Mfn2), anti-mitochondrial fission 1 (FIS1), and anti-OPA1 antibodies were from Santa Cruz Biotechnology (Santa Cruz, CA). Anti-PDK1, anti-PDH, anti-p-PDH, anti-parkin, and anti- PTEN-induced kinase (PINK) antibodies were from Abcam Biotechnology (Cambridge, MA). Anti-LC3B, mouse monoclonal anti-β-actin and horseradish peroxidase-conjugated secondary antibodies were from Proteintech (Chicago, IL). MitoSOX Red mitochondrial superoxide indicator was purchased from Invitrogen (Carlsbad, CA). RIPA lysis buffer was from Beyotime (Shanghai, China).

### Cell culture

HepG2 and HepG3B cells were cultured in DMEM medium (Gibco) supplemented with 10% fetal bovine serum (FBS; HyClone, Logan, UT), 500 U/ml penicillin, and 500 U/ml streptomycin. All cells were grown in a humidified CO2 incubator set at 37°C with 5% CO2 atmosphere.

### Cell viability assay

Briefly, we seeded approximately 8000 cells in 100 µL media into 96-well plates, and then cultured them for the indicated time. The next day, treated with various reagents for the indicated times. A total of 10 μl of 10 mg/ml MTT reagent in phosphate-buffered saline (PBS) was added for 4 h, and formazan crystals were dissolved in 150 μL dimethyl sulfoxide. The optical density at 570 nm was recorded by an enzyme-linked immunosorbent assay reader after the plate was shaken for 5 min. Each sample measurement was repeated at least three times.

### Analysis of glycolysis

After cell attachment, the medium was replaced with fresh DMEM containing 10% FBS with or without 40 or 80 mM DCA, and the cells were cultured for 24 h. Media from the dishes were collected, and Lactate production were measured using commercial colorimetric kits according to the manufacturers' protocols. The lactate kit was from Jiancheng Bio (Nanjing, China).

### Immunoblotting

Whole-cell lysates were prepared and quantified according to standard protocols, separated by sodium dodecyl sulfate-polyacrylamide gel electrophoresis, and electrophoretically transferred to Immun-Blot polyvinylidene difluoride membranes. The membranes were blocked in blocking buffer and incubated with primary antibodies and peroxidase-conjugated secondary antibodies. The bands were visualized using Pierce ECL Western Blot Substrate (Thermo Scientific, Waltham, MA).

### Inhibition of PDK1 by shRNA

Short hairpin (sh) RNA targeting PDK1 and non-targeting shRNA (shNC) were purchased from GenePharma (Shanghai, China). The PDK1 shRNA sequences were listed below: PDK1 shRNA 1: 5'-CTT-CGG-ATC-AGT-GAA-TGC-TTG-3', shRNA 2: 5'-GGT-CTC-TAG-TTT-ATG-CTG-TAT-3', and the non-target shRNA (NC) sequence was 5'-GTT-CTC-CGA-ACG-TGT-CAC-GT-3'. After adhering, cells were transfected with the shRNA plasmid using transfection reagents (Thermo Fisher Scientific, MA,USA) according to the manufacturer's protocol. After 48 h, cells were collected for the indicated assays.

### Flow cytometry

Cell were harvested and stained with Annexin V-FITC and propidium iodide (PI) (Annexin V Apoptosis Detection Kit, BD Pharmingen, USA) to measure cellular apoptosis. The MMP was determined using JC-1 dye contained within the Mitochondrial Membrane Potential Assay Kit (Beyotime). MitoSOX™ Red Mitochondrial Superoxide Indicator (Invitrogen) was used to detect ROS in the mitochondria. Analysis was performed using a BD Accuri C6 flow cytometer (Becton Dickinson, Franklin Lakes, NJ).

### Cell cycle analysis

Cells were seeded in 6-well plates, followed by indicated treatments. They were then harvested and fixed in cold 70% (*v/v*) ethanol for at least 2 h. Fixed cells were washed with PBS and stained in the dark with a solution containing 10 μg/mL PI, 0.1% Triton X-100, and 100 μg/mL RNAse for 20 min at room temperature. The DNA content was analyzed using a BD FACSCanto II flow cytometer and data analysis was performed using FlowJo software (TreeStar Inc).

### Oxygen consumption rate analysis

Cells (8×10^4^) were seeded in 96-well plates and allowed to adhere overnight. The following day, different concentrations of drugs were added into the indicated wells. Each treatment was repeated in three wells. The oxygen consumption rate was measured using oxygen-sensitive time-fluorescent probes (Mito-Xpress, Luxcel Bioscience, Cork, Ireland).

### Fluorescent staining

The production of mitochondrial ROS was evaluated by staining cells with MitoSox for 30 min at 37°C. After washing with PBS, samples were observed using an IX71 fluorescence microscope (Olympus, Tokyo, Japan).

### Fluorescence microscope

Cells were plated on glass cover slips in a 24-well plate and treated as indicated. Cells were washed in PBS, fixed with 4% paraformaldehyde for 10 min, mounted in DMEM medium with MitoTracker on standard microscope slides, then observed on an Echo-lab Revolve microscope (California, USA). For morphometric analysis, the lengths of mitochondrial were measured using ImageJ software.

### Mouse experiments

Eight female BALB/c-nude mice aged 35±41 days and weighing approximately 15 g were purchased from the Animal Experimental Center (Beijing, China). A total of 3 × 10^6^ HepG2 cells were subcutaneously injected into the upper flank. After 8 days, the nude mice were randomized into two groups (n = 4/group) when the tumor volume reached approximately 100 mm^3^, and were given the following treatments intraperitoneally (i.p.) every other day for a total of 24 days: 0.1 mL of 0.9% NaCl (control group) or 100 mg/kg DCA (DCA group). The body weights and tumor volumes of each mouse were monitored every other day until sacrifice (on day 24 after the initial treatment). All animal experiments were approved by the Animal Welfare and Ethics Group of the Laboratory Animal Science Department, Jilin University (Changchun, China).

### Immunohistochemistry

Mouse tumor tissues were fixed in 4% (w/v) paraformaldehyde, dehydrated in graded ethanol, and embedded in paraffin. Samples were then cut into 3-μm sections using a Leica microtome. Staining for TUNEL, p-PDH, PGC-1α, and lactate dehydrogenase A (LDHA), was carried out according to the manufacturers' instructions. Sections were analyzed using an inverted fluorescence microscope (Olympus).

### Citric acid assay

Mouse tumor tissues were obtained immediately after sacrifice, and then citric acid levels were measured using a commercial colorimetric kit according to the manufacturer's protocol (Jiancheng Bio).

### Statistical analysis

Data were expressed as the mean ± SD. Statistical significance between two groups was calculated by the Student's t-test using SPSS v12.0 software. *P<0.05 **P<0.01 and ***P<0.001 were considered statistically significant. All experiments were repeated at least three times.

## Results

### Inhibition of PDK1 by DCA treatment decreases cell proliferation and induces cell cycle arrest and cell apoptosis

**Figure 1:**
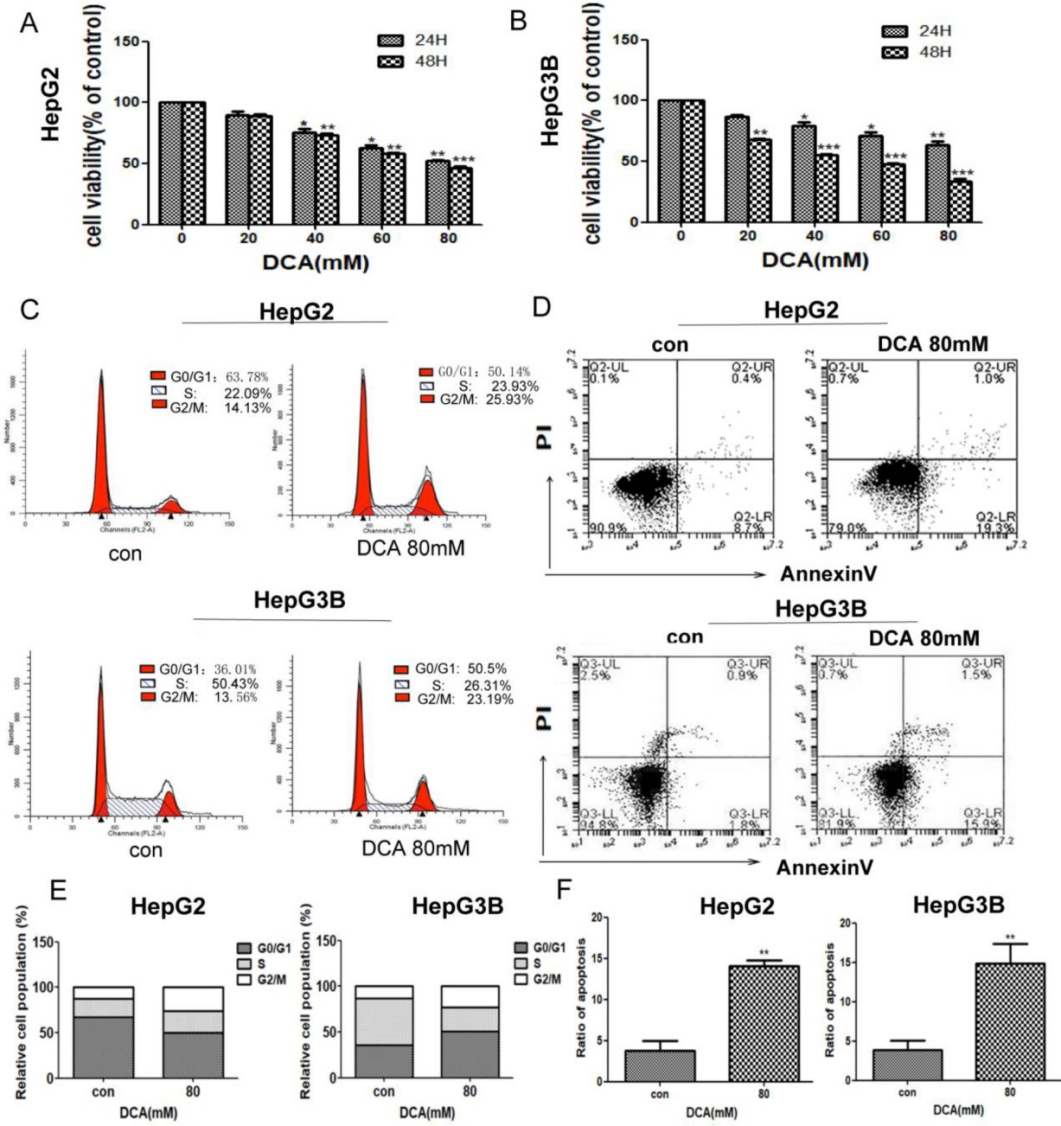
** DCA inhibits cell proliferation, arrests the cell cycle, and induces apoptosis.** (A-B) HepG2 and HepG3B cells were treated with dichloroacetate (DCA) at 0, 20, 40, 60, or 80 mM for 24 h and 48 h and then tested by MTT assay. Cells exposed to DCA at the indicated doses for 24 h were stained with propidium iodide (C) and Annexin V/PI labeling (D), then subjected to flow cytometry analysis. Total cell death and the cell cycle were quantified by flow cytometry (E-F). Data are the mean ±SD, n=3, *P<0.05, **P<0.01, ***P<0.001 compared with respective controls.

To investigate the effects of PDK1 inhibition on liver cancer, we first examined the viability of HepG2 and HepG3B cells after treatment with PDK1 inhibitor-DCA for 24 h and 48 h.The results showed that, DCA reduced the viability of HepG2 and HepG3B cells in a dose-dependent manner (Fig. 1A-B). To further examine the proliferative capacity, we examined the cell cycle of HepG2 and HepG3B cells after treatment with DCA. As shown in Fig. 1C and E, the cell cycle in HepG2 and HepG3B cells were arrested at the G2/M stage in the DCA-treated group after 24 h. Lastly, we detected apoptosis by annexin/PI staining after DCA treatment for 24h.The apoptosis rates of the 80 mM DCA HepG2 and HepG3B cell groups were higher than those of their respective control groups (Fig. 1D and F). These results showed that DCA decreases proliferation and induces cycle arrest and apoptosis in HepG2 and HepG3B cells.

### Inhibition of PDK1 induces a metabolic shift from glycolysis to oxidative phosphorylation

**Figure 2:**
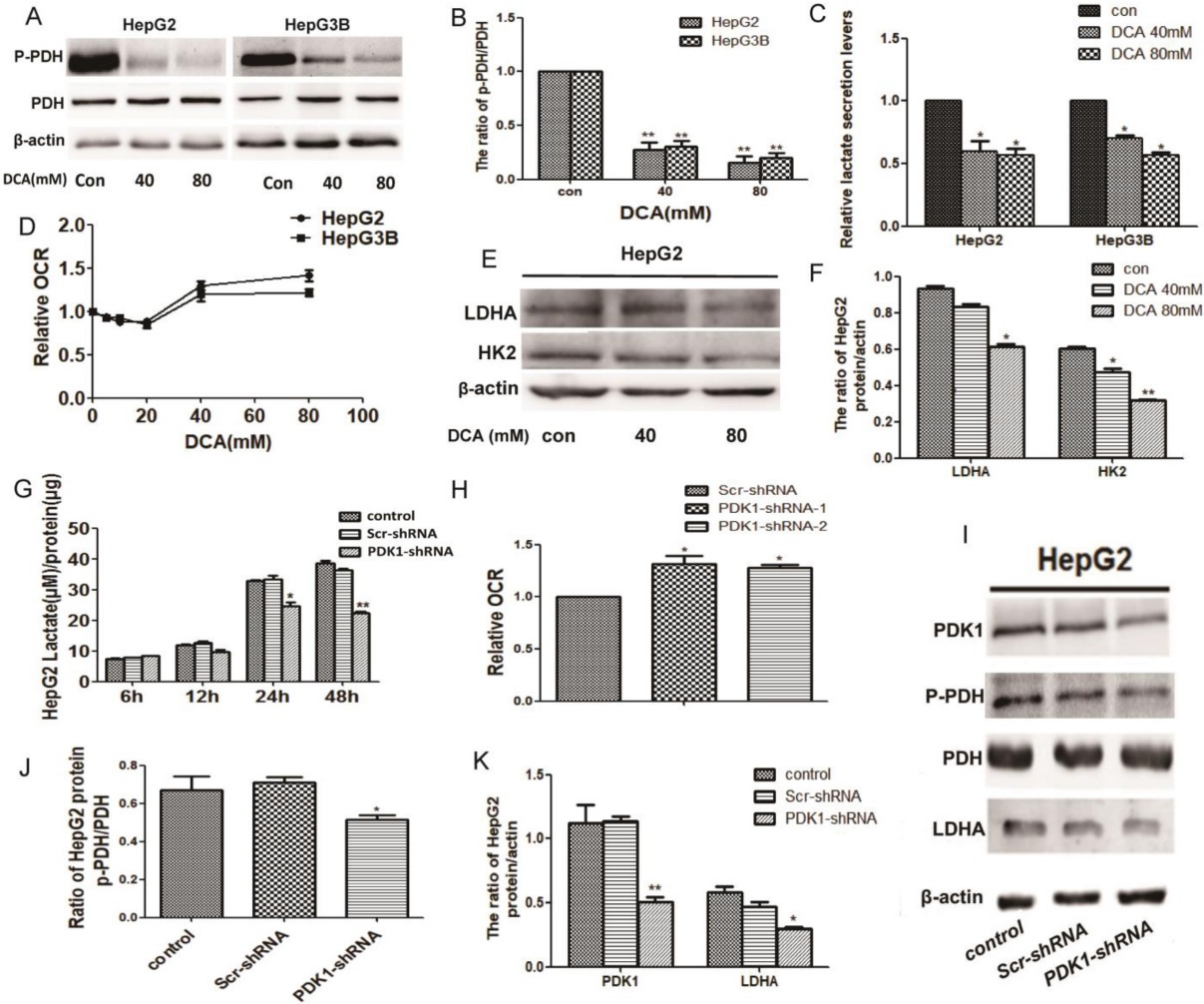
** DCA treatment or shPDK1 induce a metabolic shift from glycolysis to oxidative phosphorylation.** HepG2 and HepG3B cells were treated with DCA for 24 h, western blot analysis the levels of p-PDH and PDH (A and B), lactate concentrations were determined in the culture media and normalized to protein amounts (C) .The oxygen consumption rates of 6h were measured in HepG2 and HepG3B cells in the presence of DCA (D). (E-F) HepG2 cells were treated with DCA at the indicated doses for 24 h, equal amounts of proteins were used for western blotting to determine the levels of LDHA and HK2. HepG2 cells were transiently transfected for 48 h with the shPDK1 expression vector or empty vector, and lactate concentrations were measured after incubation for 6, 12, 24, or 48 h under basal conditions and normalized to protein amounts production (G) , the oxygen consumption rate were measured (H), western blot analysis the levels of PDK1 ,p-PDH, PDH and LDHA (I-K). Data are the mean ± SD, n=3, *P<0.05, **P<0.01 compared with respective controls.

Given that PDK1 is the key factor of glycolysis, to understand the modulating effect of PDK1 inhibition on glucose metabolism, we detected glycogen-related factors. We first detected the expression of p-PDH, PDH, HK2 and LDHA after DCA treatment or shPDK1. As shown in Fig. 2A-B, 2E-F and I-K, decreased expression of p-PDH/PDH, LDHA and HK2 were detected after DCA treatment or shPDK1.Then, a lower lactate secretion was also detected in the culture medium after PDK1 inhibition (Fig. 2C and G). To further investigate the oxidative phosphorylation level, we examined oxygen consumption rate (OCR) and an increased OCR were detected after DCA treatment for 6h or shPDK1 (Fig. 2D and H). Our results showed that inhibition of PDK1 switches cellular glucose metobolism in HepG2 and HepG3B cells.

### Inhibition of PDK1 induces oxidative damage

**Figure 3:**
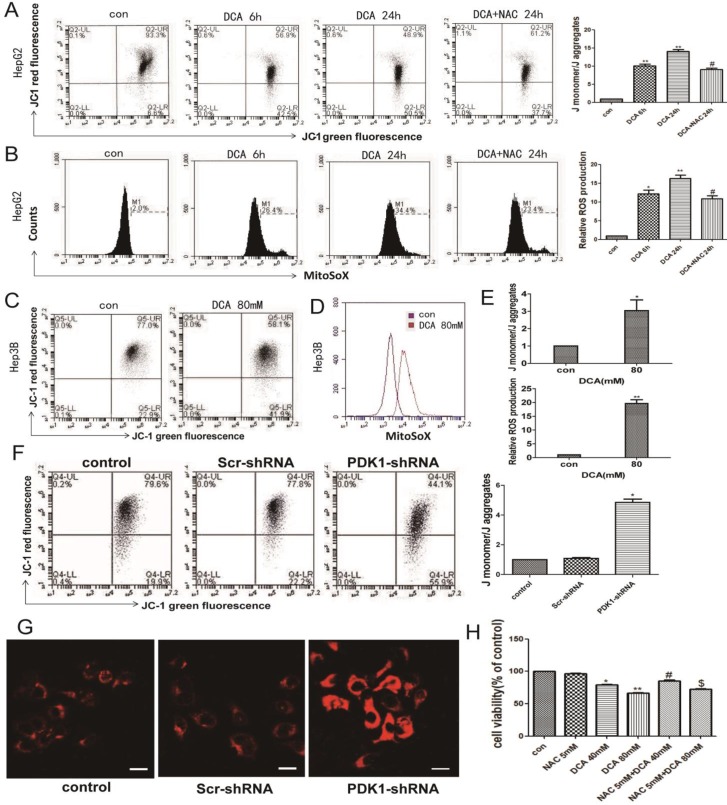
** DCA or shPDK1 reduces the mitochondrial membrane potential and increases mitochondrial ROS.** (A-B) HepG2 cells were treated with 80mM DCA for 6 h and for 24 h with or without pretreated with 5mM NAC for 1h, then assayed for the mitochondrial membrane potential with JC-1 and mtROS with MitoSOX by flow cytometry:the green fluorescence represents depolarized mitochondrial (J-monomer), and the red fluoresence represents the hyperpolarized mitochondrial (J-aggregates). The depolarization of Δψm is indicated by the increase in the ratio of J monomer/J aggregate. Data are the mean ± SD, n=3, *P<0.05, **P<0.01 compared with respective controls, ^#^*P*<0.05 compared with DCA 24 h. (C-E) The mitochondrial membrane potential and mtROS were measured in HepG3B cells in the presence of DCA for 24 h. HepG2 cells were transiently transfected for 48 h with the shPDK1 expression vector or empty vector, mtROS was measured in HepG2 cells treated as indicated (magnification ×200)(G), and the mitochondrial membrane potential with JC-1 by flow cytometry (F). (H) Cell viability was determined by MTT assay in the presence of DCA with or without NAC pretreated. Data are the mean ± SD, n=3, *P<0.05, **P<0.01 compared with respective controls, ^#^*P*<0.01 compared with DCA 40mM, ^$^*P*<0.01 compared with DCA 80mM.

Normal OXPHOS activity, stable ROS and mitochondrial membrane potential are key factors of mitochondrial pathway apoptosis. Therefore, we next examined the mitochondrial membrane potential and the levels of mitochondrial ROS. As shown in Fig. 3A-B and F-G, a decreased membrane potential and an increased mtROS were observed after DCA treatment for 6 h and 24 h or shPDK1 in HepG2 cells. The same phenomena were also seen in HepG3B cells after treatment with DCA for 24 h (Fig. 3C-E). To further verify that oxidative stress injury may induce mitochondrial dysfunction, NAC, a nonspecific antioxidant, was shown to attenuate the effects of DCA by significantly suppressing mtROS (Fig. 3A) and increasing MMP (Fig. 3B). What's more, NAC observably preserved the cell viability in HepG2 cells treated with DCA (Fig. 3H). These results showed that increasing mtROS may induce the opening of the mitochondrial permeability transition pore, leading to the oxidative damage of mitochondrial.

### The effect of PDK1 inhibition on mitochondrial quality control

**Figure 4:**
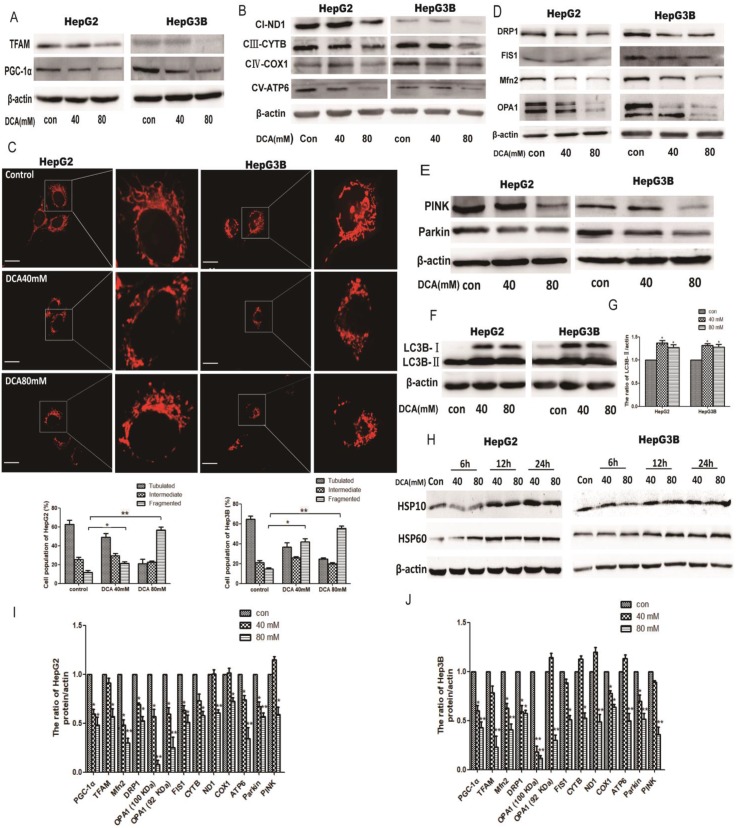
** The effect of DCA on mitochondrial quality control.** HepG2 and HepG3B cells treated with DCA were incubated for 24h. The expression of PGC-1α, Tfam (A)and mitochondrial DNA-encoded major subunits in respiratory chain complexes: ND1, CYTB, COX1, and ATP6 (B) were measured by western blot analysis. (C) The mitochondrial network of HepG2 and HepG3B cells after treatment with DCA were determined by staining and flurescent microscopy (400×). The proportion of cells (n=100 cells for each sample) with tubulated, intermediate and fragmented mitochondrial was quantified. Western blot analysis for DRP1, FIS1, Mfn2, OPA1 (D), and PINK, parkin (E) and LC3B (F-G) in cells treated as indicated. (I-J) Quantitation of the ratio of the indicate proteins. Data are the mean ± SD, n=3, *P<0.05, **P<0.01 compared with respective controls. (H) Western blot analysis for HSP10 and HSP60 in HepG2 and HepG3B cells treated with DCA at different time points as indicated.

**Figure 5:**
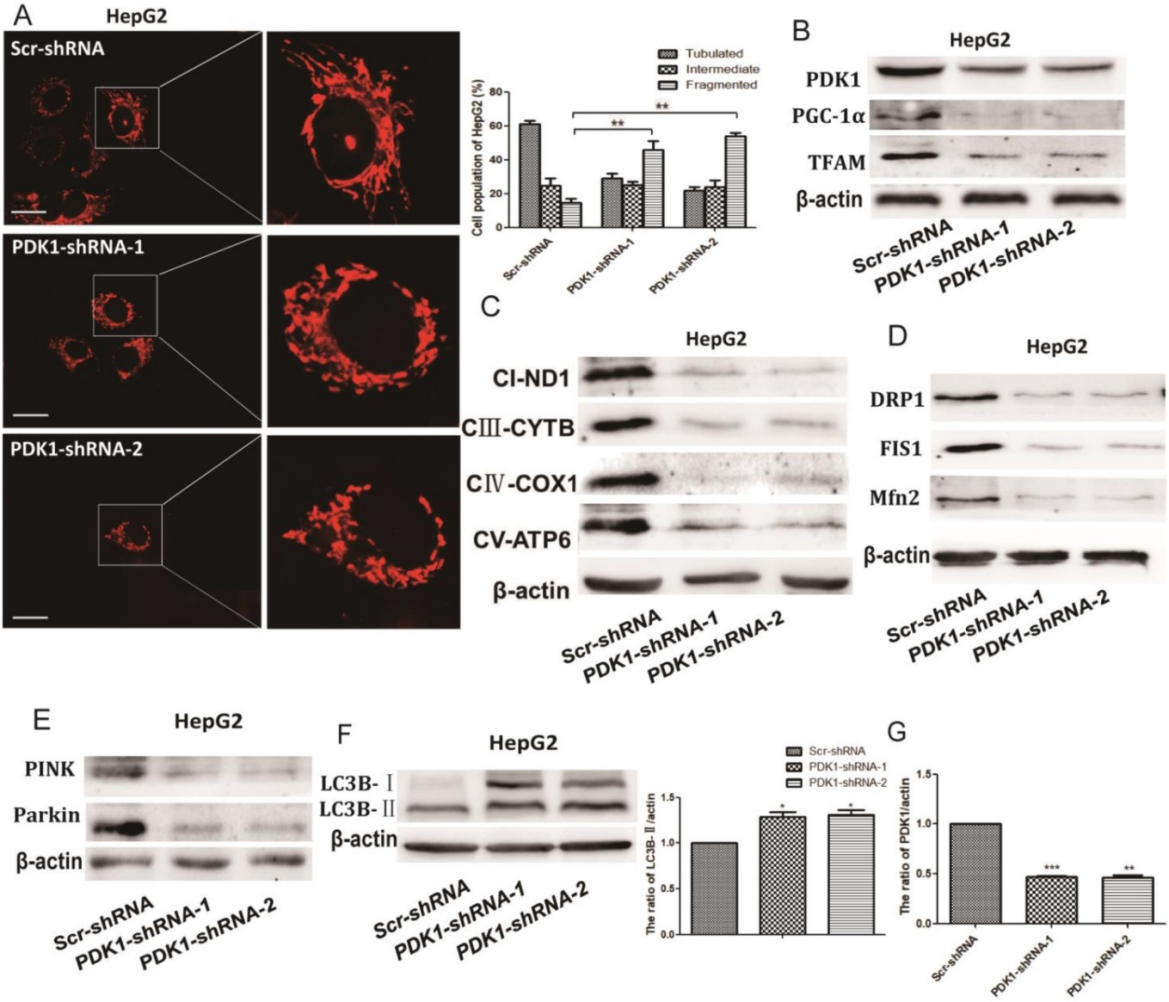
** The effect of shPDK1 on mitochondrial quality control.** HepG2 cells were transiently transfected with the shPDK1 expression vector or empty vector for 48 h. (A) The mitochondrial network were determined by staining and flurescent microscopy (400×). The proportion of cells (n=100 cells for each sample) with tubulated, intermediate and fragmented mitochondrial was quantified. Western blot analysis of PDK1, PGC-1α and Tfam (B) mitochondrial DNA-encoded major subunits in respiratory chain complexes: ND1, CYTB, COX1, and ATP6 (C), DRP1, FIS1and Mfn2 (D), PINK, parkin (E) and LC3B (F) after treatments. (G) Quantitation of the ratio of PDK1. **P<0.01, ***P<0.01compared with respective controls.

As quality control of mitochondria plays an important role in maintaining mitochondrial oxidative phosphorylation, especially the levels of mitochondrial biosynthesis. Then, we explored the key factors of mitochondrial biosynthesis protein-PGC-1α. As shown in Fig. 4A and 5B, the expression of PGC-1α and the downstream molecule Tfam were reduced after DCA treatment or shPDK1. Similar results were obtained for the expression of mitochondrial DNA-encoded major subunits in respiratory chain complexes (Fig. 4B and 5C). Next, we explored the effect of PDK1 inhibition on mitochondrial morphology. Cells in the control group tended to have long mitochondria that contained interconnected filaments. As the DCA concentration increased, the mitochondrial network contained shorter and more fragmented filaments, the same change was seen after shPDK1, which was shown by a significant increase in the percentage of cells with fragmented mitochondrial (Fig. 4C and 5A). We detected the expression of mitochondrial fusion and fission proteins, and found that OPA1, Mfn2, FIS1, and DRP1 protein levels were all decreased by DCA treatment or shPDK1 (Fig. 4D and 5D). Mitochondrial dynamics are closely related to mitochondrial autophagy, so molecules of mitophagy were examined. Western blotti ng revealed decreased levels of Parkin and PINK (Fig. 4E and 5E). We also detected the autophagy marker LC3B-Ⅱ, and found increased expression after DCA treatment or shPDK1 (Fig. 4F and 5F). Moreover, we examined UPR^mt^ proteins in HepG2 and HepG3B cells, and observed that the expression of HSP10 and HSP60 was increased after treatment with DCA in time- dependent manner (Fig. 4H).

### DCA attenuates tumor growth and induces glucose metabolic and mitochondrial quality control changes in HepG2 cell xenografts

**Figure 6:**
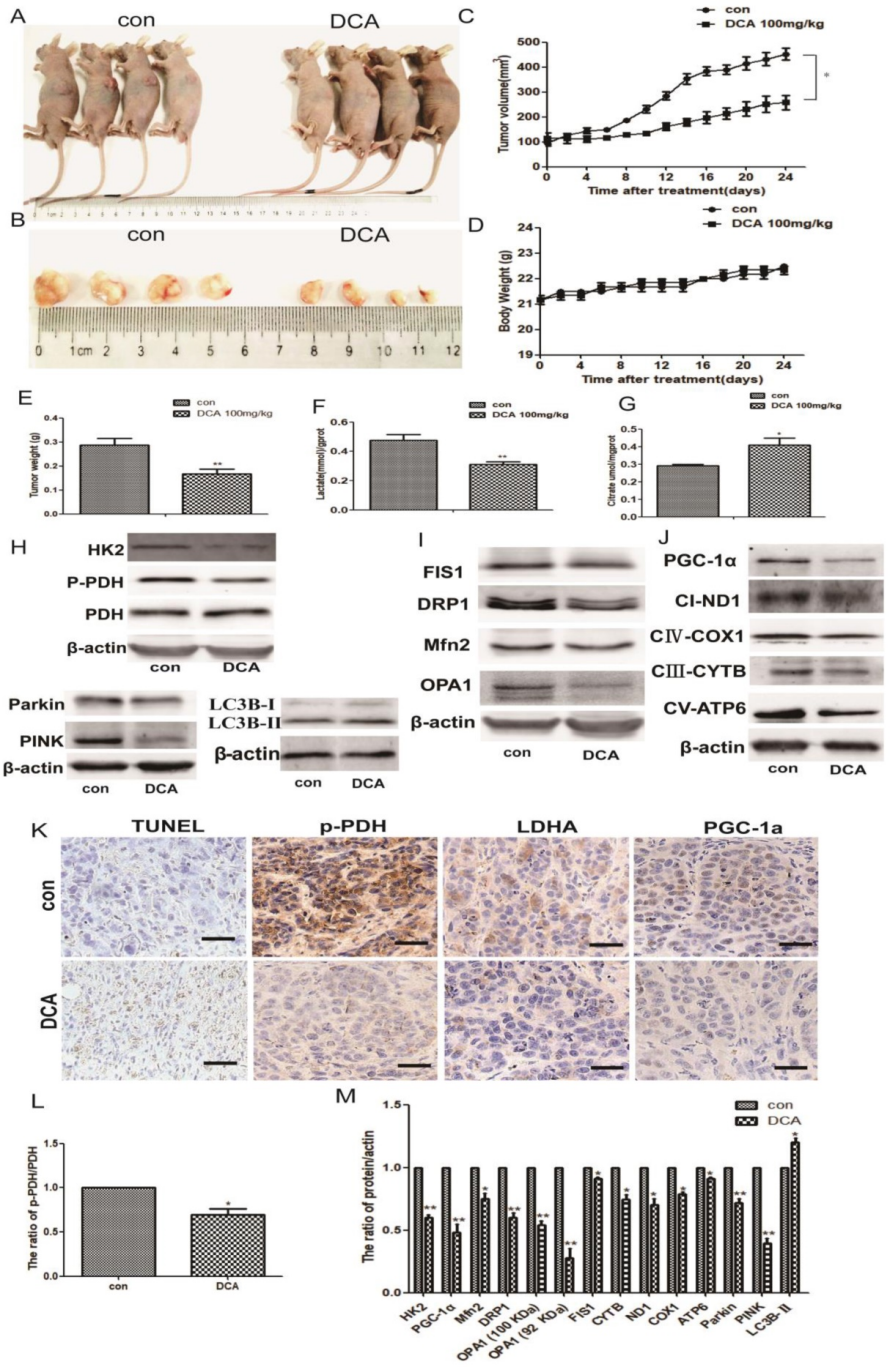
** DCA suppresses tumor growth in a subcutaneous xenograft.** (A-B) Images of xenograft tumors formed in nude mice injected with normal saline (control group) and the DCA-treated group. (C) Body weight gain profiles of vehicle- and DCA-treated nude mice. (D and E) Tumor volume and weights were calculated from measurements. (F-G) Lactic acid in the serum and citric acid in the tissues of nude mice were measured. Data are the mean ± SD, n=4, *P<0.05, **P<0.01 compared with controls. (H-J and L-M) PDH, p-PDH, HK2, OPA1, Mfn2, FIS1, DRP1, parkin, PINK, LC3B, PGC-1α, ND1, COX1, CYTB, and ATP6 levels in xenografts were detected by immunoblotting. Data are the mean ± SD, n=3, *P<0.05, **P<0.01 compared with controls. (K) Immunohistochemistry of TUNEL, p-PDH, PGC-1α, and LDHA in HepG2 cell xenografts. TUNEL staining revealed obvious apoptosis in DCA-treated nude mice (bar, 100 μm).

To confirm the potential anticancer role of DCA via the inhibition of PDK1 *in vivo*, we tested its therapeutic efficacy in subcutaneous human HepG2 xenografts. Tumor-bearing nude mice were randomly divided into two groups: control and DCA (100 mg/kg) intraperitoneally treated groups. After 24 days, we measured the tumor volume and weight of HepG2 xenografts at sacrifice. Tumors treated with DCA were significantly smaller and weighed less than tumors in the control group (Fig. 6A-C and E). There was no significant difference in gross toxicity or body weight between the two groups (Fig. 6D).

Next, we tested serum lactic acid levels and the citric acid content of nude mouse tissues. Decreased lactic acid and increased citric acid were detected in tumor-bearing mice compared with the control group (Fig. 6F-G), similar to the *in vitro* results. We also examined the levels of metabolic and mitochondrial quality control-related proteins. Western blotting showed increased LC3b-Ⅱ expression, and decreased expression of PDH, p-PDH, HK2, OPA1, Mfn2, FIS1, DRP1, parkin, PINK, PGC-1α, NADH dehydrogenase subunit (ND1), cytochrome c oxidase subunit 1(COX1), cytochrome B (CYTB), and mitochondrially encoded ATP synthase membrane subunit 6 (ATP6) (Fig. 6H-J and L-M).

Lastly, we detected apoptosis in HepG2 xenografts using the TUNEL staining assay, and staining for p-PDH, PGC-1α, and LDHA. Apoptosis and the proliferation ratio were higher in the DCA-treated group than in the control group, while p-PDH, PGC-1α, and LDHA expression was lower than in the control group (Fig. 6K). These results indicated that DCA inhibited tumor growth, induced a metabolic shift from glycolysis to oxidative phosphorylation, and changed the mitochondrial quality control *in vivo*.

## Discussion

The glycolysis phenotype, also known as the Warburg effect, is a ubiquitous phenomenon in most cancers, where glucose uptake and glycolysis occur at a high rate while mitochondrial respiration is suppressed 29, 30. The PDK1/PDH loop has an important role in regulating glycolysis and mitochondrial oxidative phosphorylation energy metabolism 31. Therefore, restoration of mitochondrial oxidative phosphorylation by PDK1 inhibition becomes a potential new therapeutic target for tumor treatment 31-33. However, the mechanisms of how changes in metabolic patterns affect tumor cell mitochondrial function are unclear. Mitochondrial have developed several mechanisms that act to survey and maintain organelle homeostasis, especially the mitochondrial protein quality control to attenuate buildup of misfolded proteins in the organelle 34, 35. Therefore, the role of mitochondrial quality control between glucose metobolism reprogramming and mitochondrial function becomes the focus of our research.

In the present study, we performed *in vitro* and *in vivo* experiments and found that DCA significantly inhibited the proliferation of HepG2 and HepG3B cells and promoted apoptosis, while effectively inhibiting the growth of xenografts in nude mice. Further gene silencing of PDK1 in HepG2 cells revealed that knocking down PDK1 also promoted apoptosis. OXPHOS is the main function of mitochondria, and Shen et al. found that DCA in glioblastoma promoted oxidative phosphorylation by inhibiting glycolysis^36^. In our experiments, the inhibition of PDK1 significantly inhibited the level of glycolysis in HepG2 and HepG3B cells, and upregulated mitochondrial-associated oxidative metabolism. This indicated that PDK1 inhibition can break the balance between glycolysis of HepG2 and HepG3B cells and mitochondrial OXPHOS.

Normal OXPHOS activity, stable ROS, and mitochondrial membrane potential are key factors in mitochondrial pathway apoptosis 27. Dey et al. found that ROS reduced the mitochondrial membrane potential and participated in apoptosis resistance in osteosarcoma 37. Therefore, it may be a therapeutic advantage to restore the mitochondrial function of tumor cells. Mitochondrial OXPHOS activity is coupled with the mitochondrial membrane potential 38, 39. We found that DCA or shPDK1 significantly altered the redox balance of HepG2 and HepG3B cells, resulting in excess ROS production and decreased the membrane potential. NAC, a nonspecific antioxidant, was shown to attenuate the effects of DCA by significantly increasing MMP while scavenging mtROS, and observably preserved the cell viability in HepG2 cells treated with DCA. Therefore, as a by-product of the mitochondrial respiratory chain, ROS can affect the mitochondrial membrane potential to promote tumor cell apoptosis, as well as inducing abnormal protein expression and dysfunction. Whether metabolic reprogramming induced by PDK1 inhibition leads to other mitochondrial reactions or changes the mitochondrial function is still unclear. Therefore, we next explored the specific mechanism involved in the effects of metabolic changes on mitochondrial function.

Mitochondrial quality is a prerequisite for function. Mitochondria may activate a number of mass mechanisms to maintain homeostasis 40, 41. We divided mitochondrial quality control into the following pathways: 1) mitochondrial network morphology, including fusion and fission; 2) mitochondrial biosynthesis, including PGC-1a and ROS; and 3), mitochondrial UPR reaction, mitochondrial autophagy, and redox reaction.

First, the network structure of mitochondria is constantly undergoing fission and fusion, which is necessary for maintaining mitochondrial function. Pajuelo-Reguera et al. found that DCA altered the mitochondrial network structure^28^. In our experiments, the mitochondrial network morphology of HepG2 and HepG3B cells gradually condensed and exhibited shorter and more fragmented filaments after DCA treatment or shPDK1. We related this mitochondrial network restructuring to changes in FIS1, DRP1, OPA1, and Mfn2 protein levels. This indicated that the PDK1 inhibition induced downregulation of mitochondrial fusion and fission altered the mitochondrial morphology, which is consistent with a study by Griparic et al. who found that deletion of the fusion protein OPA1 was accompanied by an increase in the mitochondrial height of the fragmentary structure^42^, ^43^. Second, PGC-1α, the main protein of mitochondrial biosynthesis, regulates the expression of proteins involved in mitochondrial fusion and autophagy 12, 44. We therefore speculated that the shPDK1 or DCA-induced decreased expression of PGC-1α may be responsible for mitochondrial fission and autophagy downregulation. Concurrently, Tfam and its regulated mitochondrial complex protein were also decreased after PDK1 inhibition, which represents mitochondria damage. The same protein change was also observed in transplanted tumors in nude mice. This suggested that PGC-1α plays an important role in linking glucose metabolism and mitochondrial quality control. Third, Hayners et al. demonstrated that the upregulation of mitochondrial HSPs protected impaired mitochondrial function and homeostasis^45^. In our experiments using prolonged DCA treatment, we found that the mitochondria of tumor cells initiated UPR protection to maintain protein homeostasis in mitochondria.

It was recently shown that PINK1/Parkin is involved in a pathway to regulate mitochondrial quality via the degradation of dysfunctional mitochondria by mitophagy 11, 46, 47. Tanaka et al. reported that mitochondrial autophagy was accompanied by a decrease in membrane potential that affected mitochondrial morphology 21. In our experiments, DCA or shPDK1 significantly increased the expression of autophagy marker LC3B-Ⅱ, enhanced autophagy leads to the degration of Parkin and PINK. Therefore, mitochondrial autophagy cannot completely remove damaged mitochondrial, which aggravated stress damage. These indicating that the inhibition of PDK1 affected mitochondrial autophagy, which in turn aggravated stress damage, consistent with *in vivo* protein experiments.

Thus, the inhibition of PDK1 induced mitochondrial quality control disorders, and PGC-1α played an important role among it.

In conclusion, our experiments demonstrate that changes in metabolism by the inhibition of PDK1 affect mitochondrial quality control in tumor cells. Mitochondrial oxidative phosphorylation has an important role in tumor cell chemotherapeutic drug sensitivity, while the regulation of mitochondrial proteins and organ levels is one of the main factors determining mitochondrial function.

## Abbreviations

DCA: Dichloroacetic acid
OXPHOS: oxidative phosphorylation
PDK1: Pyruvate dehydrogenase kinase 1
MMP: mitochondrial membrane potential
ETC: electron transport chain
ROS: reactive oxygen species
PGC1α: peroxisome proliferator-activated receptor gamma coactivator 1α
TFAM: Mitochondrial transcription factor A
